# Supplementary material for: Mycoplasma Pneumoniae bronchiolitis and hypoxemia: A retrospective cohort study on risk and prognosis
Source: PLoS One. 2025 Oct 30;20(10):e0335604. doi: 10.1371/journal.pone.0335604 (PMC12574826; doi:10.1371/journal.pone.0335604)
Supplement: S1 Table — (DOCX) [file pone.0335604.s001.docx]

Supplementary Table 1 Clinical data, laboratory indicators and imaging data of children with BO sequelae

| Project | Patient No. 1 | Patient No. 2 | Patient No. 3 | Patient No. 4 | Patient No. 5 | Patient No. 6 | Patient No. 7 |
| --- | --- | --- | --- | --- | --- | --- | --- |
| Gender | Male | Female | Male | Female | Male | Female | Female |
| Age (year) | 3.3 | 3.5 | 2.2 | 3.5 | 8.9 | 3.3 | 7.7 |
| Fever duration (day) | 6 | 5 | 6 | 3 | 5 | 5 | 4 |
| Fever severity | High fever | High fever | High fever | High fever | High fever | High fever | High fever |
| Cough duration (day) | 4 | 6 | 4 | 5 | 8 | 3 | 4 |
| Wet rales | - | + | + | - | + | + | + |
| Wheezing sounds | + | + | + | + | + | + | + |
| History of allergic diseases | Allergic rhinitis | Allergic rhinitis | Allergic rhinitis | Allergic rhinitis | Allergic rhinitis, asthma | Allergic rhinitis | Allergic rhinitis, asthma |
| Aeroallergen test | Dust mites | Dust mites, Cockroaches | Dust mites, Mold | Dust mites, Cat dander | - | Dust mites | Dust mites, Cat dander, Dog dander |
| Food allergen test | Shrimp | - | Milk, Beef | - | Beef, Crab, Shrimp | - | Milk |
| Leukocyte (×10^9^/L) | 11.3 | 5.4 | 7.6 | 5.7 | 7.1 | 5.8 | 13.1 |
| CRP (mg/L) | 43.5 | 33.5 | 14.2 | 64.4 | 50.4 | 60.4 | 46.4 |
| LDH (IU/L) | 627.3 | 426.7 | 841.2 | 486 | 667.7 | 511.9 | 542 |
| Ferritin (μg/L) | 712 | 486 | 593 | 617 | 799 | 655 | 825 |
| D-dimer (mg/L) | 2.72 | 0.72 | 1.12 | 1.23 | 1.39 | 2.52 | 2.54 |
| MP DNA load (copy/ml) | 79000 | 9400 | 71000 | 49000 | 510000 | 77000 | 210000 |
| Macrolide resistance mutation | + | + | + | + | + | + | + |
| Infected lung lobes | 5 | 4 | 4 | 5 | 6 | 5 | 5 |
| Time from symptom onset to treatment initiation | 4 | 4 | 3 | 3 | 4 | 3 | 4 |
| Doxycycline usage | + | - | - | - | + | + | + |
| Routine doses of methylprednisolone | + | + | + | + | + | + | + |
| Pulse doses of corticosteroids | - | - | - | - | - | - | - |

+ Present; - Absent
